# Microbial community composition predicts bacterial production across ocean ecosystems

**DOI:** 10.1093/ismejo/wrae158

**Published:** 2024-08-06

**Authors:** Elizabeth Connors, Avishek Dutta, Rebecca Trinh, Natalia Erazo, Srishti Dasarathy, Hugh Ducklow, J L Weissman, Yi-Chun Yeh, Oscar Schofield, Deborah Steinberg, Jed Fuhrman, Jeff S Bowman

**Affiliations:** Scripps Institution of Oceanography, UC San Diego, La Jolla, CA 92037, United States; Scripps Polar Center, UC San Diego, La Jolla, CA 92037, United States; Department of Geology, University of Georgia, Athens, GA 30602, United States; Savannah River Ecology Laboratory, University of Georgia, Aiken, SC 29802, United States; Lamont-Doherty Earth Observatory, Columbia University, New York, NY 10964, United States; Scripps Institution of Oceanography, UC San Diego, La Jolla, CA 92037, United States; Scripps Institution of Oceanography, UC San Diego, La Jolla, CA 92037, United States; Lamont-Doherty Earth Observatory, Columbia University, New York, NY 10964, United States; Department of Biological Sciences, University of Southern California, Los Angeles, CA 90089, United States; Department of Biology, The City College of New York, New York, NY 10003, United States; Department of Biological Sciences, University of Southern California, Los Angeles, CA 90089, United States; Coastal Ocean Observation Laboratory, Institute of Marine and Coastal Sciences, School of Environmental and Biological Sciences, Rutgers University, New Brunswick, NJ 08901-8520, United States; Virginia Institute of Marine Science, College of William & Mary, Gloucester Point, VA 23062, United States; Department of Biological Sciences, University of Southern California, Los Angeles, CA 90089, United States; Scripps Institution of Oceanography, UC San Diego, La Jolla, CA 92037, United States; Scripps Polar Center, UC San Diego, La Jolla, CA 92037, United States

**Keywords:** microbial ecological function, community structure, bacterial production, random forest regression

## Abstract

Microbial ecological functions are an emergent property of community composition. For some ecological functions, this link is strong enough that community composition can be used to estimate the quantity of an ecological function. Here, we apply random forest regression models to compare the predictive performance of community composition and environmental data for bacterial production (BP). Using data from two independent long-term ecological research sites—Palmer LTER in Antarctica and Station SPOT in California—we found that community composition was a strong predictor of BP. The top performing model achieved an *R*^2^ of 0.84 and RMSE of 20.2 pmol L^−1^ hr^−1^ on independent validation data, outperforming a model based solely on environmental data (*R*^2^ = 0.32, RMSE = 51.4 pmol L^−1^ hr^−1^). We then operationalized our top performing model, estimating BP for 346 Antarctic samples from 2015 to 2020 for which only community composition data were available. Our predictions resolved spatial trends in BP with significance in the Antarctic (*P* value = 1 × 10^−4^) and highlighted important taxa for BP across ocean basins. Our results demonstrate a strong link between microbial community composition and microbial ecosystem function and begin to leverage long-term datasets to construct models of BP based on microbial community composition.

## Introduction

Microbial ecosystem functions, defined as microbial activity at the community scale, are an essential component of Earth’s biogeochemical cycles, including carbon and nitrogen [1, 2]. Typically measured via a stable isotope tracer or via enzymatic activity, microbial functions are often—but not always—strongly linked to microbial community composition [1]. Previous work has identified strong links between community composition and various components of the carbon and nitrogen cycles and demonstrated that community composition data can be used to make quantitative predictions of some functions [3–9]. For instance, because microbial community composition strongly influences decomposition and respiration rates in soil [10], bacterial community composition can be used to predict dissolved organic carbon concentrations in leaf litter [4]. Identifying the connections between microbial composition and function is of utmost importance in the context of global change, where microbial diversity loss is predicted to increase with unknown consequences to microbial function and subsequently carbon and nitrogen cycling [11].

In one example of a microbial ecosystem function, marine heterotrophic bacteria (here meaning heterotrophic members of the bacteria and archaea) incorporate phytoplankton-derived dissolved organic matter (DOM) into new bacterial biomass through bacterial production (BP). This repackaging of DOM into microbial biomass is a key step in the microbial loop, where organic matter is recycled to the higher trophic levels via bacterivory by protists [12]. As the abundance and productivity of bacteria thus rely on the availability of phytoplankton-derived DOM, BP is strongly related to primary production (PP), with an average global BP:PP ratio of ~10% [13, 14]. However, this ratio of BP:PP is highly variable (~0.5%–25%) depending greatly on the time and space scales analyzed [14, 15].

Here, we leveraged long-term time series of BP and other microbial and environmental data across two coastal regions to construct predictive models and better understand the links between microbial community composition and BP. The selected long-term study sites were the Palmer Long-Term Ecological Research site along the western Antarctic Peninsula (wAP) and the long-running San Pedro Ocean Time Series (SPOT) located in the San Pedro Channel in Southern California. Unfortunately, many long running time series do not regularly measure BP, which can be prohibitive or difficult to measure in some settings as it requires a radioactive tracer. The Palmer Long-Term Ecological Research (LTER) site off the western Antarctic Peninsula (wAP) is a good example; there have been hundreds of measurements for bacterial community composition since 2015 with a concurrent BP measurement only 24% of the time. However, previous work has shown that BP is strongly related to bacterial community composition and bacterial abundance along the wAP [3, 16]. These works showed that community composition and bacterial abundance were the two most important variables in a linear model that best described BP over five spring–summer seasons along the wAP [3]. Other work showed that increasing BP coincided with a change in community composition during the 2014 summer season [17]. A more robust dataset of BP is necessary as (along with PP) BP has the most direct effect on the production and consumption of dissolved organic carbon and nitrogen in the upper ocean of the western Antarctic Peninsula [18].

In this study, we utilized the random forest algorithm to construct and compare several models that predict BP from bacterial community composition using data from the Palmer LTER along the wAP and from Station SPOT in Southern California, where more observations of BP are available. This approach has previously been shown to be effective for predicting biogeochemical standing stocks that are strongly influenced by microbial processes [4, 19]. We expand on previous studies by comparing our amplicon models of BP to one constructed from only environmental and fluorescence data, demonstrating that community composition may be a better predictor of BP than environmental data and highlighting a fundamental microbial composition–function relationship. Overall, our findings demonstrate a strong link between the composition of microbial communities and their ecosystem functions in two disparate coastal research sites.

## Materials and methods

### Palmer Station Long-Term Ecological Research data

**Figure 1:**
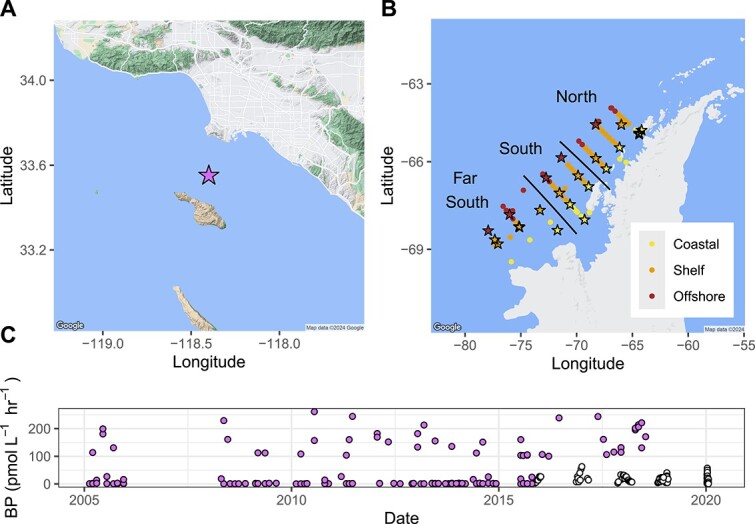
(A) Sampling location of the San Pedro Ocean Time Series off the southern California coast (SPOT, star). SPOT has a total of 133 amplicon samples from monthly samples from 2005 to 2020 with an associated bacterial production (BP) measurement (purple values in 1C). (B) Sampling locations along the Palmer LTER (PAL) grid along the western Antarctic Peninsula. The PAL dataset contains 453 amplicon samples, 108 of which have an associated bacterial production (BP, pmol leucine per L^−1^ h^−1^) measurement (24%, black stars). (C) Timing and amplitude of the total 241 observations of BP used in this study (purple from SPOT, white from PAL). Underlying map courtesy of Google Maps.

Palmer Long-Term Ecological Research Project (PAL) amplicon data used in this study were downloaded from the NCBI SRA database under BioProject PRJNA901488. A minority of the samples used in this study (113 samples, 25%) were collected weekly from 10 m depth at PAL Station B (lat: −64.774167, long: −64.0544) over austral summer seasons from 2015 to 2020 (PAL samples, November–March, Fig. 1C). The majority (340 samples, 75%) of samples were collected during two cruises, aboard the ASRV *Laurence M. Gould* (LMG) in January of 2019 and 2020, respectively (LMG1901 and LMG2001 samples), and from the small vessel *Hadar* on 7 March 2020 (PD2001). Samples collected during the two LMG cruises were collected along a sampling grid of stations 10 km apart arranged in 10 onshore to offshore lines spaced 100 km apart along the Peninsula and opportunistically along the ship track throughout the nine LTER subregions (along-shore regions of offshore, shelf, coastal regions; cross-shore regions of north, south, and farther south, Fig. 1B) extensively outlined in previous work [20, 21].

For the PAL amplicon samples, 1 L of seawater was filtered through a sterile 0.2 μm Supor membrane disk filter (Pall Corporation, Port Washington, NY, USA) and stored at −80°C until extraction. Filters were extracted using the KingFisher Flex Purification System and MagMax Microbiome Ultra Nucleic Acid Extraction kit (ThermoFisher Scientific, Waltham, MA, USA). Extracted DNA was sent to Argonne National Laboratory for amplicon library preparation and sequencing using the MiSeq platform (Illumina) with the universal primers 515F and 806R [22], and a 2 × 151 bp library architecture. Illumina reads were then filtered, denoised, and merged with dada2 [23].

BP samples were collected alongside amplicon data for 108 of the 453 samples (24%) used in this study. PAL BP data were downloaded from the ERRDAP database [24]. All samples were collected and processed according to PAL LTER standard protocols using radioactively labeled 3H-leucine [15].

Bacterial abundance data via flow cytometry were also downloaded from the PAL LTER ERRDAP database [24] and collected alongside amplicon data for all 453 samples (100%). Flow cytometry samples were prefiltered (with a Coring Falcon 40 μm Cell Strainer) before running on an AccuriC6 flow cytometer (BD Biosciences, Franklin Lakes, NJ, USA) equipped with a blue (488 nm) laser. All samples were stained and incubated in the dark for 15 min with the nucleotide stain SYBR Green 1 (Molecular Probes, Inc., Eugene, OR, USA) and the manufacturer’s recommended concentration. Quality control for absolute cell counts were confirmed by spiking 10 μl of 1:2500 diluted 1 μm Fluoresbrite Yellow Microspheres (Polyscience Inc., Fishers, IN, USA) to each sample. All samples were run on “slow” with a flow rate of 14 μl min^−1^ for 1 min and measured for forward scatter, side scatter, and green emission (488/533 nm excitation/emission).

Bacterial populations were identified using a self-organizing map (SOM) from forward scatter, side scatter, and green emission following previous methods [3, 25]. In brief, a training set was constructed with data from five randomized sample days, with one from each year. These data were trained using a toroidal map with a grid size of 41 × 41 using the “kohonen” package in R [26]. Populations were identified using *k*-means clustering and *k* = 6 was chosen through a priori knowledge of populations and the visual evaluation of a within-cluster sum of squares scree plot. This *k*-means cluster model was then used to classify events in all flow cytometry samples. HNA and LNA bacterial populations were identified from the flow cytometry clusters and were converted into cells mL^−1^. These two bacterial populations were then combined to form a total cell count (bacterial abundance in cells mL^−1^) for each sample. Total cell count outliers (two observations) were removed when their values were outside the range Q1–1.5 × (Q3 − Q1), Q3 + 1.5 × (Q3 − Q1), where Q1 and Q3 are the first and third quartiles, respectively.

### San Pedro Ocean Time Series data

Amplicon data from the upper 200 m were downloaded from the EMBL database under accession PRJEB48162 and processed following developed protocols [27]. This dataset includes monthly measurements at the San Pedro Time Series (SPOT, Fig. 1A) off the coast of California from 2005 to 2018 for community composition, via amplicon sequencing of two distinct filter size classes (0.2–1 and 1–80 μm). Once retrieved from the database, amplicon sequences were trimmed with cutadapt [28], split into 16S rRNA gene reads using bbtools [29] and denoised and merged with dada2 [23]. For each sampling day, the distinct filter size classes were combined (via simple addition of the absolute read counts of each ASV in both size fractions) for our final analysis to better match sequences with no filter size classes from the PAL dataset.

BP samples were collected alongside amplicon data for all 133 monthly samples (100%) used in this study [30]. All samples were collected and processed according to SPOT standard protocols using radioactively labeled 3H-leucine [31]. Finally, environmental data were also collected for all SPOT samples, including NO_3_, PO_4_, and CTD measurements for temperature, oxygen, salinity, and fluorescence following standard protocols [27, 30]. These environmental variables were used to create the SPOT-ENV model.

### 16S rRNA gene amplicon data

PAL and SPOT data were QC’d and denoised to amplicon sequence variants (ASVs) with dada2 [23] following previously published procedures [19]. Because different primers were used for these datasets the ASVs are consistent only with each dataset. PAL and SPOT ASVs data were then analyzed with paprica v0.7.1 [32]. Paprica utilizes phylogenetic placement with Gappa [33] EPA-ng [34] and Infernal [35] to place query reads on a reference tree constructed from the full-length 16S rRNA genes from all completed genomes in GenBank [36]. All unique reads are assigned to both internal branches (closest estimated genomes, see paprica documentation for further description) and if possible, terminal branches (closest completed genomes) on the reference tree. Once assigned, unique reads that were assigned as mitochondria or chloroplasts were omitted, as well as any reads that only appeared once (25% of all ASVs). The phylogenetic placement approach that is inherent to paprica results in ASV aggregation by phylogenetic edges. This edge-level data unified the data across primers and were used for relative mean maximal growth rate calculations and joint model construction.

The latest version of paprica includes a prediction for relative mean minimal doubling time from codon usage patterns adapted from the R package gRodon [37]. To make doubling time predictions, paprica applies gRodon by calculating the relative mean minimal doubling time on each completed genome in the paprica database. Because the goal is to estimate the theoretical maximum growth rate based on genetic signature, predictions are presented only in relative terms without correction for temperature. Mean minimal doubling times are then assigned to reads according to their point of placement on the paprica reference tree. Placements to terminal branches (i.e. closest completed genomes) are assigned the rate corresponding with that genome, placements to internal branches (i.e. closest estimated genomes) are assigned the average of all rates of terminal nodes belonging to that clade. A predicted relative mean minimal doubling time for the community is calculated by taking the average of the rates assigned to all edges.

### Random forest regression modeling

All random forest regression models were created using the “randomForest” package in R [38]. For each model, samples were restricted to those that had an observed BP (BP_obs_) measurement. Those samples with a BP_obs_ were further randomly separated into training (80%) and validation (20%) datasets. A random forest regression was run on the training dataset and then used to predict BP (BP_pred_) for the validation dataset. Model performance was assessed using residual mean square error (RMSE) and *R*^2^ values for the validation dataset. The optimal number of decision trees (ntree in randomForest, the point where more trees did not improve model performance) was set to 500 and number of variables to randomly sample as candidates at each split (mtry in randomForest, where too few variables can lead to overly biased results and too many is inefficient) was set to 10 after random forest hypertuning over the range of 100–1200 ntree and 1–20 mtry; where ntree = 500 and mtry = 10 produced the highest *R*^2^ in a linear regression of BP_obs_ and BP_pred_ for the validation data.

### Palmer Long-Term Ecological Research Project absolute abundance calculation and model input variables

For the PAL data only, we were able to leverage the available flow cytometry data to produce an absolute abundance for each of the closest completed genome matches from the amplicon reads. We multiplied relative abundance of quality controlled unique 16S rRNA gene reads—corrected for 16S rRNA gene copy number by paprica—by the total bacterial abundance (as stated above, the HNA and LNA combined cell count from flow cytometry). For PAL random forest models only, we compared a model with absolute abundance as input (PAL-CCG) to a model with relative abundance as input (PAL-CEG). As flow cytometry data were not available for SPOT, only relative abundance data were used in the SPOT random forest model (SPOT-CEG) and for the joint model (JOINT-CEG) with data from both PAL and SPOT, with an additional categorical variable for region. The fifth and final model was created with only the environmental data from Station SPOT (SPOT-ENV) as input. A PAL-ENV model was not created as there were not enough environmental data available.

### Feature selection on input variables with Boruta

All models included a feature selection step before the random forest regression model. The ASV relative or absolute abundances were reduced by the Boruta feature selection algorithm [39]. This algorithm finds those variables that contribute most to model performance by iteratively removing those variables for which randomization does not diminish model performance. For SPOT-ENV, feature selection did not reduce the number of variables in the model (as all were deemed relevant).

### Cross validation of random forest and predictions of bacterial production

For each of the five random forest models we tested (PAL-CEG, PAL-CCG, SPOT-CEG, SPOT-ENV, and JOINT-CEG), a final model was then trained with all samples with BP_obs_ and we validated our results via random forest cross validation performed with the package “rfUtilities” [40]. This is an independent assessment of model performance that provides the average mean standard error and average variance explained with 10% of the data omitted randomly over 99 iterations of the model. Out best performing model, the cross-validated JOINT-CEG model, was then used to predict BP for those samples where BP data were missing (346 PAL samples). For statistical comparisons of BP_pred_ and BP_obs_ across multiple spatial scales, *P* values were adjusted for multiple comparisons via the Holm–Bonferroni method. In addition to predicting BP, the increase in mean standard errors of BP predictions because of each ASV being randomly shuffled was calculated (% IncMSE). A high % IncMSE indicates a taxon that was important for model performance.

## Results

### Trends in observed bacterial production, abundance, and community composition

**Figure 2:**
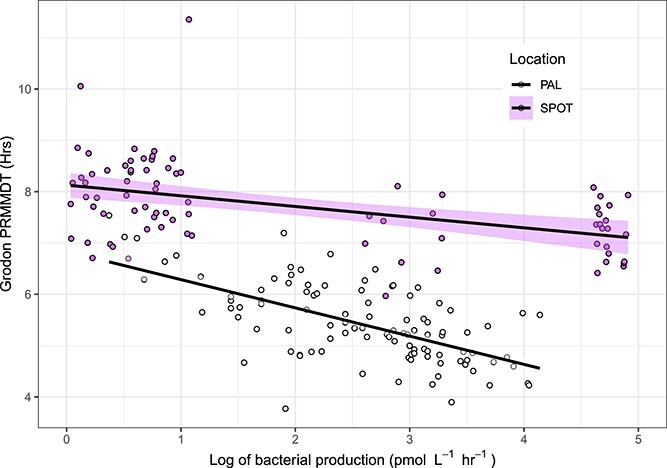
Relationship between log of observed BP (pmol leucine per L^−1^ hr^−1^) and predicted relative mean minimal doubling time (PRMMDT) from gRodon for both the San Pedro Ocean Time Series (SPOT, *R*^2^ = 0.30) and palmer LTER (PAL, *R*^2^ = 0.49).

Rates of observed BP (BP_obs_) ranged from 0.42 to 62.70 pmol L^−1^ hr^−1^ across all sampling locations and years at PAL (Fig. 1C). In the cruise samples (LMG1901, LMG2001, and PD2001) BP_obs_ from the surface (0 m) and above the 50 m mixed layer samples were significantly higher than samples below 50 m (Kruska–Wallis adj *P* value = 6.29 × 10^−5^). BP_obs_ showed no significant trends across-shore (Coast/Shelf/Offshore) or alongshore (North/South/Far South, see Fig. 1B) for the LTER grid (Kruskal–Wallis adj *P* values = 0.13 and 0.45, respectively). At SPOT, rates of observed BP (BP_obs_) ranged from 0.07 to 261.36 pmol L^−1^ hr^−1^ across all years (Fig. 1C). Observed BP was negatively correlated to minimum doubling time estimated by gRodon for both SPOT and PAL samples (Fig. 2, *R*^2^ = 0.30 and *R*^2^ = 0.49, respectively).

PAL cell abundance ranged from 7.43 × 10^3^ to 6.91 × 10^5^ cells ml^−1^ across all sampling sites and years. In the cruise samples (LMG1901, LMG2001, and PD2001) total cell abundances from the surface (0 m) were significantly higher than samples from the mixed layer and below 50 m (Kruskal–Wallis adj *P* value = 6.60 × 10^−16^). Cell abundances showed no significant trends across-shore (Coast/Shelf/Offshore) or alongshore (North/South/Far South) the LTER grid (Kruskal–Wallis adj *P* values = .19 and .78, respectively), and cell abundance was significantly correlated with observed BP (linear regression *R*^2^ = 0.18, *P* value = .013).

PAL community composition (the relative abundance of unique ASVs) varied significantly over the sampling locations in a nonmetric multidimensional scaling (NMDS) of the square root of Bray–Curtis distances of Hellinger-transformed relative abundance. An ANOSIM analysis of station samples indicated significant differences in community composition across years (ANOSIM R statistic = 0.07, *P* value = .002) and months across years (i.e. all January data binned together, ANOSIM R statistic 0.17, *P* value = .001). For 2019 and 2020 cruise samples, both alongshore and across-shore stations sites had statistically different community compositions (alongshore N/S/Far S: ANOSIM R statistic 0.02, *P* value .001; across-shore C/S/O: ANOSIM R statistic = 0.06, *P* value = .001).

### Comparing random forest model performance

**Figure 3:**
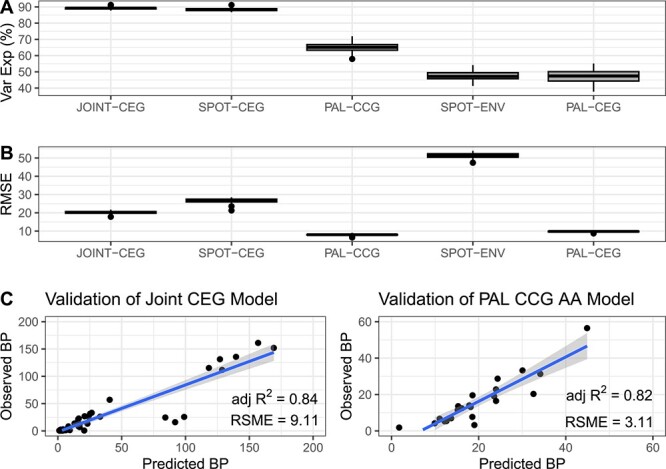
Comparison of cross validation of random forest models where (A) is the percent variance explained (% Var Exp) of each of the models and (B) is square root of the mean square error (RMSE) of each of the models over their cross validation (*n* = 99, with 10% of data withheld). The Joint-CEG model is built from the relative abundance of closest estimated genomes (CEG) of amplicon data from both the San Pedro Ocean Time Series (SPOT) and Palmer LTER (PAL) for all samples with an observation of BP (*n* = 241) and location (“PAL” or “SPOT”). This model is compared to one each of CEG from each site (SPOT-CEG model and PAL-CEG model), from environmental variables at SPOT (SPOT-ENV), and a model built from absolute abundance (relative abundance multiplied by bacterial cell count) of closest completed genomes from PAL (PAL-CCG). Individual models from PAL performed more poorly than the joint model in cross validation, and the environmental model from SPOT performed the worst (with the lowest percent variance explained and highest RMSE). (C) are validation data from the two best performing models, the Joint-CEG and PAL-CCG model.

The Joint-CEG model performed best both in our initial model testing (Table 1) and in cross validation when compared to models from the two regions on their own (Fig. 3). BP_pred_ from the random forest regression models matched BP_obs_ from observed samples with high fidelity when testing each of the five models (testing 20% of samples, adj *R*^2^ are listed in Table 1). BP_pred_ and BP_obs_ matched while initially training the model for comparison (80% of samples, adj *R*^2^ are listed in Table 1) and while training the final model (100% of samples). Cross validation of the final random forest regression model indicated a high average variance explained and a low average square root of the mean square error for each of the models (Fig. 3). The Joint-CEG model and PAL-CCG model performed the best in cross validation, while the environmental model from SPOT performed the worst (with the lowest percent variance explained and highest RMSE in Fig. 3). For a direct comparison of the environmental model to our best performing model, we created a Joint-CEG model with the same number of variables (The six taxa with the highest %IncMSE). This truncated Joint-CEG model performed very similarly to the Joint-CEG (In cross validation, % Var Exp = 82.2 and RSME = 19.1).

**Table 1:** Random forest model performance statistics in our initial model validation (20%) and median cross validation (10% of the data, *n* = 99) for the five models compared in this study.

Model name	Training data (80%) adj *R*^2^	Validation data adj *R*^2^	Training data (100%) adj *R*^2^	Median cross-validation permuted % Var Exp	Median cross-validation RMSE
JOINT-CEG	0.65	0.84	0.98	89.3	20.2
SPOT-CEG	0.87	0.93	0.98	88.2	26.8
SPOT-ENV	0.64	0.32	0.32	47.2	51.4
PAL-CEG	0.76	0.57	0.94	47.4	9.81
PAL-CCG	0.96	0.82	0.96	65.9	7.87

### Palmer Long-Term Ecological Research Project BP_pred_ from the Joint-CEG model

**Figure 4:**
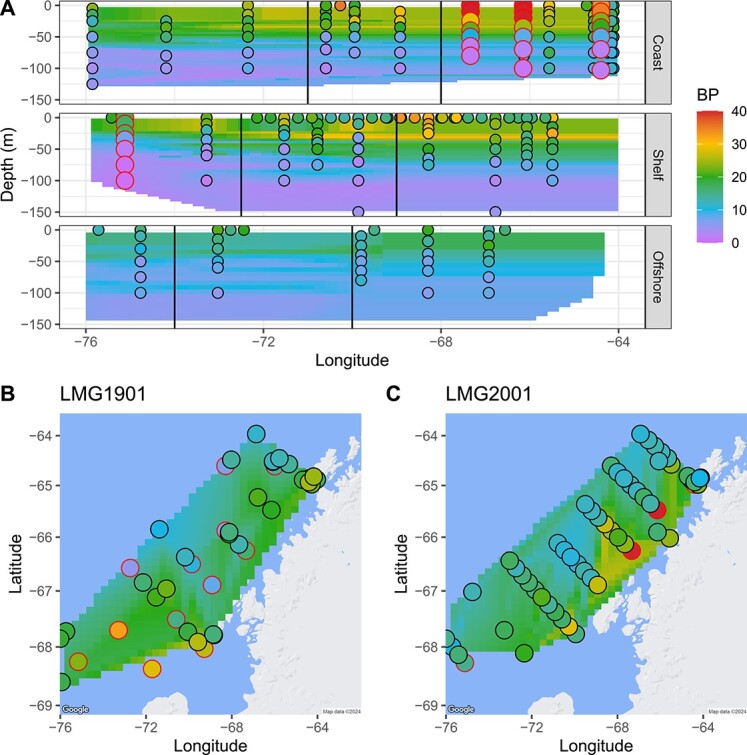
Joint model bacterial production (BP, pmol leucine per L^−1^ hr^−1^) predictions expand our understanding of Palmer LTER (PAL) BP across the surface PAL LTER 2019 (LMG1901) and 2020 cruises (LMG2001) and depth profiles from LMG2001 and Palmer Deep (PD2001) cruises. Joint model predictions of BP where it is missing in PAL dataset (with linear interpolation behind) closely match observations of BP.

BP_pred_ ranged from 1.09 to 40.17 pmol L^−1^ hr^−1^ incorporated leucine over all sampling locations and years (Fig. 4). In the cruise samples (LMG1901, LMG2001, and PD2001) BP_pred_ from the surface (0 m) and above the 50 m mixed layer samples were significantly higher than samples below 50 m (Kruskal–Wallis adj *P* value = 6.60 × 10^−16^). BP_pred_ showed no significant trends alongshore the LTER grid (Kruskal–Wallis adj *P* value = .30, North/South/Far South, see Fig. 1B). However, offshore measurements were significantly lower than coastal and shelf measurements in the across-shore comparison (Wilcox rank sum test adj *P* value of Offshore vs. Shelf = 8.29 × 10^−3^). Finally, we used a Mantel test to examine geospatial correlation for BP_obs_ and BP_pred_. A geospatial matrix of sample latitude, longitude, and depth was not significant when compared to BP_obs_**(***P* value = .093) but was significant for all BP (BP_obs_ & BP_pred_*P* value = 1.0 × 10^−4^). Finally, BP_pred_ from the Joint-CEG model was correlated to BP_pred_ from the PAL-CCG model (linear regression *R*^2^ = 0.76, *P* value = 2.20 × 10^−16^).

### Important taxa for predicting bacterial production in both Joint-CEG and PAL-CCG

**Figure 5:**
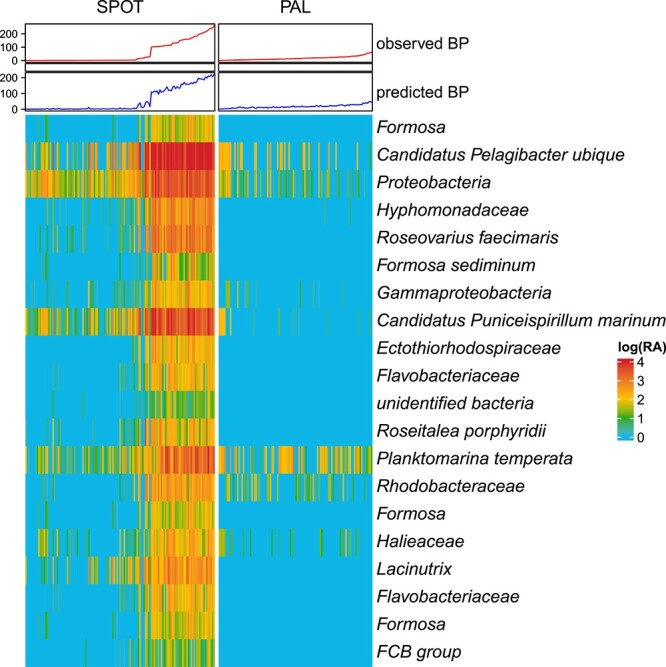
List of top twenty important taxa for the joint model (JOINT-CEG) performance, by percent increase in mean square error (%IncMSE). Heatmap is relative abundance (RA) of each of these taxa over all samples with a matching measurement of BP from the SPOT and PAL datasets. The top heatmap annotation is observed bacterial production (BP, in pmol leucine per L^−1^ hr^−1^) and predicted BP ( pmol leucine per L^−1^ hr^−1^) from the joint model, ordered from smallest to largest.

**Figure 6:**
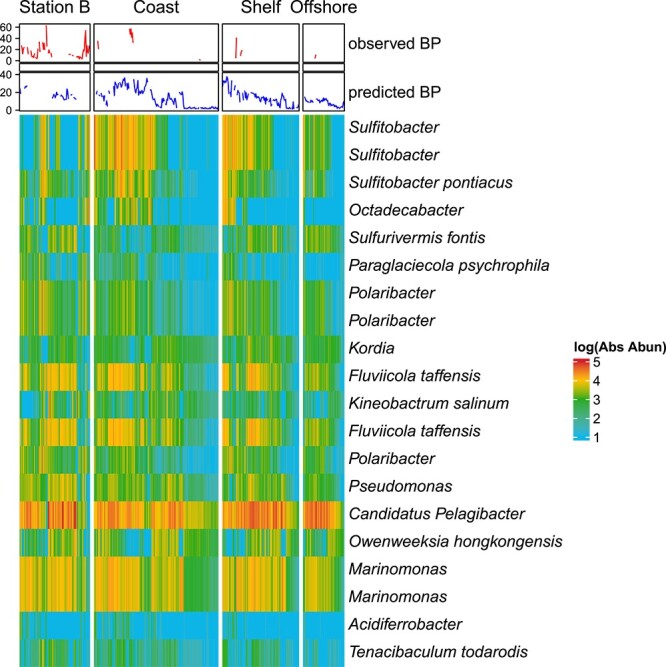
List of top twenty important taxa for the Palmer Station closest completed genome model (PAL-CCG) model, by percent increase in mean square error (%IncMSE). Heatmap is absolute abundance (Abs Abun) of each of these taxa over the PAL dataset, partitioned into LTER station locations. The top heatmap annotation is observed bacterial production (BP, pmol leucine per L^−1^ hr^−1^) and predicted BP ( pmol leucine per L^−1^ hr^−1^) from the PAL-CCG model.

Random forest regression models report the taxa (ASVs or edges from paprica) that are most important for model performance as percent increase in mean standard error (% IncMSE) when the variable is randomly shuffled. Here, we report the top 20 most important taxa in our two best performing models of BP, the Joint-CEG model (highest cross validation % Variance Explained) and the PAL-CCG model (lowest cross-validation RSME). The top 20 most important taxa from the Joint-CEG random forest model (Fig. 5, with every % IncMSE listed in Supp Table 1) had values that ranged from 555.8% (*Formosa*) to 72% (FCB Group). For this model, only 8 of the top 20 most important taxa had a relative abundance in PAL samples (for instance, *Formosa* is not present in any PAL samples). In SPOT samples with higher BP_obs_ and BP_pred_ than any of the PAL data (<100), the relative abundance of the two most important taxa (which mapped to *Formosa* and *Pelagibacter ubique*) were also high, along with *Puniceispirillum marinum* and *Planktomarina temperata*. In the second best performing model, PAL-CCG model (Fig. 6), the top 20 most important taxa ranged from 70% IncMSE (*Sulfitobacter* spp.) to 2.1% (*Tenacibaculum todarodis*)*.*

## Discussion

The application of machine learning–based models in the field of microbial ecology is steadily increasing, including models that can predict diseases caused by microorganisms [41], species interactions [42], and even biogeochemical processes [19]. In our study, we leveraged large datasets of observations from the Palmer LTER and from the SPOT to expand our understanding of how microbial communities lead to specific biogeochemical outcomes. Because community composition data were comparatively common and easy to collect, models that quantitatively link community composition with ecophysiology parameters can greatly improve our understanding of the distribution of rates and standing stocks and can suggest microbial mechanisms (i.e. shifts in taxon abundance) underlying changes across space and time. We also demonstrated that it is possible to create a single model that predicts rates with good accuracy across regions.

Though we included only two regions here, our results suggest that it is possible to build a single global model that can predict BP from community composition for any region included in the training data. Finally, in our study, all four models built from community composition data outperformed a model built from environmental data. On a regional scale, our best performing model allowed us to predict values where measurements were missing and to determine abundance trends in taxa that are most important for predicting BP. The increased data density from our predictions allowed us to make conclusions about the distribution of BP across the western Antarctic Peninsula study region that were not possible from BP_obs_ alone.

Our input variables for the PAL random forest regression model—measurements for bacterial abundance via flow cytometry and bacterial community composition via 16S rRNA gene sequencing—follow similar patterns as previously reported for Palmer LTER data [3]. Bacterial abundance from 2003 to 2014 ranged from 1 × 10^3^ to 4 × 10^6^ cells ml^−1^, with higher abundances in coastal waters as in this study [43]. Community composition data from previous studies show similar significant differences across sampling months—with notable community changes in January—and with depth in the water column [16, 44]. BP_obs_ are also within the range of most data from previous years (majority 0–60 pmol L^−1^ hr^−1^ from 2003 to 2014) where higher production was measured in inshore regions [43].

We saw significant geospatial trends in BP when we included BP_pred_ in our analysis, highlighting the potential for gap-filling biogeochemical data with observations of microbial community composition. Our model would be improved by additional measurements of community composition, bacterial abundance, and BP_obs,_ when BP_obs_ is anomalously high (> 100 pmol L^−1^ hr^−1^ in previous years), which are currently unrepresented in our training data from PAL (highest value of 60 pmol L^−1^ hr^−1^). Even with that limitation, our random forest regression model greatly outperformed linear models on similar Palmer LTER data in previous years [3] at predicting BP. This comes with the caveat that it might not be possible to determine predictive taxa at all sites from a joint model, as some of the predictive data from the joint model seems to have no relationship with BP in PAL (see *Planktomarina temperate* in Fig. 5).

Our comparison of BP to predicted relative mean minimal doubling time (Fig. 2) demonstrated a significant negative correlation between average minimum doubling time and BP. The differences in predicted relative mean minimal doubling time across the two sites may demonstrate ecological differences, where the SPOT bacterial community is always primed for fast growth, while at Palmer, the bacterial community is replaced if it is not actively growing. The minimum doubling time estimated by gRodon in paprica should be treated as an incomplete measure of doubling time, given that the prediction depends on bacteria present in all available completely assembled genomes in RefSeq, where Antarctic seawater bacteria are poorly represented [45]. Improvements in the representation of Antarctic seawater bacteria in sequencing databases, the inclusion of assembled Antarctic genomes in our analysis, as well as more lab-based culture work on Antarctic bacterial growth rate would improve our estimates and our comparison. gRodon output and productivity may have a slightly noisy relationship in that gRodon always estimates a maximum and does not consider interactions between community members. Even with these caveats, it is encouraging to see a significant negative correlation between predicted relative mean doubling time and BP, as it demonstrates the inherent connection between bacterial community genetics (codon usage) and cellular processes (growth rate and BP).

Whereas our random forest regression model trained on data from both California and Antarctica was able to predict BP with success, there are definite drawbacks to the Joint-CEG Model. Foremost, it is probable that ecological differences diminished predictive power across these widely separated regions. This is well demonstrated by *Polaribacter*, a genus that is adapted to and much more abundant in colder climates [46]*.* Although it is one of the top 20 most important taxa in the PAL-CCG model (by % IncMSE, Fig. 6), it is not an important taxon for the Joint-CEG Model at all (Fig. 5). Overall, SPOT taxa dominated the Joint-CEG Model, with 60% of the top 20 taxa with representatives only at SPOT. Although the RMSE of the Joint-CEG model (20.2) was lower than the SPOT-CEG model (26.8), it was much higher than the RMSE of the PAL-CCG model (7.78), which indicates regional models are important to reduce error in predictions of BP. However, the observed model fidelity and still strong predictive power of the Joint-CEG model suggests that it may be possible to construct global models for BP if training data are drawn from an adequate number of representative regions.

Although the genus *Polaribacter* demonstrated clear ecological differences, the genus *Sulfitobacter* highlighted potential ecological similarities across the two sites. The most important taxa for the PAL-CCG model to predict BP by % IncMSE, *Sulfitobacter* had elevated abundances in samples with higher BP for both the Antarctic and Station SPOT data in California (Figs 5 and 6). In previous work, the family *Rhodobacteraceae* (the family to which *Sulfitobacter* belongs) and *Polaribacter* were the two most abundant bacteria when BP was highest during a phytoplankton bloom at Station B in Antarctica [17]. Members of the *Rhodobacteracae* were also dominant in the community when BP was the highest in a 5-year analysis of community composition along the wAP [3]. Cultured representatives of Antarctic *Sulfitobacter* have the ability to breakdown various carbohydrates, organic acids, amino acids, and peptides of phytoplankton-derived DOM [47]. In coastal California, *Sulfitobacter* can play an important role in the sulfur cycle by converting dimethylsulfoniopropinate to dimethlysulfide [48], but the extent of this process along the wAP and its relationship to BP is unknown.

Our findings suggest that machine learning methods and especially random forest regressions are important tools to understand the complex datasets inherent to microbial ecology. Random forest regression and similar techniques make it possible to predict biogeochemical rates such as BP from microbial community composition data and even compare it to predictions from environmental data. This approach provides a new technique for filling gaps in biogeochemical data sets and for making predictions where it is not practical to measure rates. By evaluating the efficacy of random forest regression models trained in one ocean biome to another, we can even determine which microbial taxa are globally vs. regionally significant for a process of interest. Our models demonstrate a strong link between microbial community composition and ecosystem functions. We anticipate that with enough training data, it will be possible to construct global BP models that captures most microbial composition–function relationships.

## Supplementary Material

Supp_Table1_wrae158

## Data Availability

All sequences are available at NCBI SRA BioProject PRJNA901488 or EMBL under accession PRJEB48162. The code for this manuscript is located on the first author’s GitHub, at https://github.com/beth-connors/wAP-predicted-bacterial-production.
